# Effects of Optical Beams on MIMO Visible Light Communication Channel Characteristics

**DOI:** 10.3390/s22010216

**Published:** 2021-12-29

**Authors:** Jupeng Ding, Chih-Lin I, Jintao Wang, Hui Yang

**Affiliations:** 1 Key Laboratory of Signal Detection and Processing in Xinjiang Uygur Autonomous Region, School of Information Science and Engineering, Xinjiang University, Urumqi 830046, China; 2 China Mobile Research Institute, Beijing 100053, China; icl@chinamobile.com; 3 National Research Center for Information Science and Technology, Department of Electronic Engineering, Beijing Tsinghua University, Beijing 100084, China; wangjintao@tsinghua.edu.cn (J.W.); huiyang@tsinghua.edu.cn (H.Y.)

**Keywords:** visible light communication, MIMO, optical beams, non-Lambertian sources, 6G mobile communications

## Abstract

Under 5G envision, for pushing visible light communication (VLC) channel model evolution to various non-Lambertian beams, this paper introduces the typical commercial non-Lambertian beams, such as Luxeon rebel and side emitter, into the conventional analytical VLC channel model. The numerical results illustrate that the non-Lambertian beams can significantly affect the VLC channel frequency response characteristics. Compared with the traditional Lambertian beam, Side Emitter optical beam could naturally bring up to about 56.8% VLC multi input multi output channel capacity deviation, which objectively opens a new discussion dimension for enhancing VLC transmission performance.

## 1. Introduction

For satisfying the explosive traffic in the evolving 5G and coming 6G era, some countries have initiated relevant research activities. For implementing new wireless technique paradigms, such as terahertz communications, visible light communications (VLC) are emphasized as candidates for green 6G network [1,2].

Up to now, VLC have been discussed and investigated for almost two decades. VLC could make full use of the advantages from solid state lighting, which is also known as light emitting diodes (LED). It should be noted that the current VLC research is almost based on Lambertian LED assumption [3,4,5,6]. Nevertheless, for rendering certain lighting performance, the secondary lens and the reflection cups are frequently attached to the original LED chips by the manufacturers. As a matter of fact, the resultant optical beams usually do not follow the conventional Lambertian pattern any more [7,8].

In VLC channel characteristics aspects, the analytical VLC channel model is widely accepted by the researchers and engineers, thanks to its quite limited computation complexity [3,4,5]. Nevertheless, the non-Lambertian VLC channel characteristics investigation is still limited within the time-consuming deterministic modeling scheme [8,9]. On the other hand, the multiple input multiple output (MIMO) techniques have been actively applied in enhancing the VLC capacity performance [10]. Similarly, the VLC channel capacity estimation is still absent for the actual non-Lambertian optical beams. It should be noted that there are many potential VLC application scenarios adopting LED sources following non-Lambertian beam characteristics. Therefore, for accurately evaluating MIMO VLC performance variation, it is essential to consider the optical beam effects on MIMO VLC channel characteristics. In this work, for the first time, the VLC analytical models are derived for two typical non-Lambertian optical beams. Accordingly, based on the above non-Lambertian channel models, the MIMO capacities for the related two beams are formulated for the following numerical estimation.

The remainder of this work is organized as follows: In Section 2 spatial optical beams model is introduced. The analytical VCL channel model for the Lambertian and the typical non-Lambertian beams are described in Section 3. In Section 4, the MIMO VLC channel capacity is presented for the above distinct optical beams. Section 5 gives the relevant numerical evaluation results. Finally, Section 6 concludes this paper.

## 2. Spatial Optical Beams Characteristics

### 2.1. Lambertian Optical Beams

**Figure 1:**
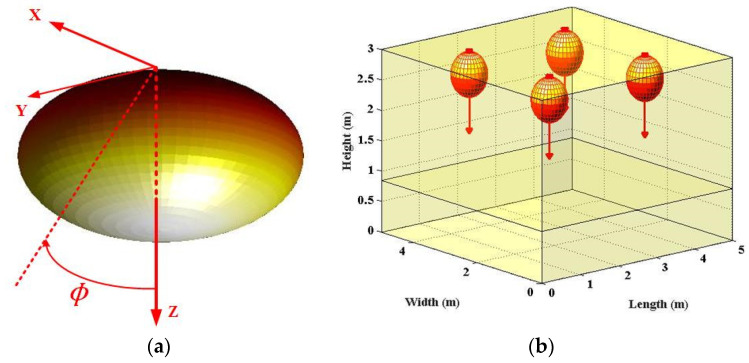
(**a**) 3D spatial optical beam of Lambertian LED; (**b**) Lambertian LED-applied indoor scenario, respectively.

In conventional VLC irradiance analysis, the LED sources are viewed as Lambertian emitters. Such processing means that radiation intensity is also one cosine function of the viewing angle and could be given as [3,4,5,6]:

(1) $$I_{\mathrm{Lam}}\left(\phi \right)=\frac{m_{\mathrm{Lam}}+1}{2\pi }\cos^{m_{\mathrm{Lam}}}\left(\phi \right)$$

where ϕ is the irradiance angle with respect to the source perpendicular axis. The Lambertian order $m_{\mathrm{Lam}}$ is given by $m_{\mathrm{Lam}}=-\mathrm{In}2/\mathrm{In}(\cos(\phi_{1/2}))$, where $\phi_{1/2}$ is the half power angle of the Lambertian beam [3,4,5,6]. Moreover, in Equation (1), $(m_{\mathrm{Lam}}+1)/2\pi$ is the normalization factor which assure the emitted all power equal to 1W from one generalized, Lambertian beam. Specifically, in Figure 1, the Lambertian optical beam and the applied typical indoor scenario are illustrated. Specifically, based on the above Lambertian beam mathematical expression, the MATLAB plot tool is utilized to draw the Figure 1.

### 2.2. Non-Lambertian Optical Beams

For realizing desired lighting performance, the actual optical beams are frequently modified by the sum of three terms: the optical refraction by the encapsulating cup, the optical reflection inside the lens and the optical reflection via the reflecting cup.

According to the reported measure results, the non-Lambertian optical beams could be numerically fitted by the linear combination of certain Lambertian functions, and some other functions generated by the mentioned reflections and refractions.

Without loss of generality, two typical non-Lambertian optical beams are included in this work. Specifically, both optical beams are derived from the LUXEON Rebel LED and Side Emitter LED, respectively.

**Figure 2:**
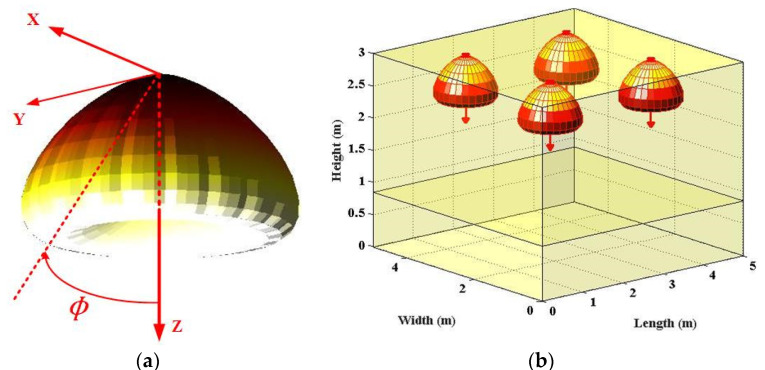
(**a**) 3D spatial optical beam of LUXEON Rebel non-Lambertian LED; (**b**) LUXEON Rebel non-Lambertian LED-applied indoor scenario, respectively.

For the LUXEON Rebel case, the spatial radiation intensity could be profiled by one sum of two Gaussian functions [7]:

(2) $$I_{\mathrm{LUX}}(\phi )=\sum_{i=1}^{2}g_{{{}_{1}}_{i}}^{{}_{\mathrm{LUX}}}\exp[-\ln2{\left(\frac{\left|\phi \right|-g_{{{}_{2}}_{i}}^{{}_{\mathrm{LUX}}}}{g_{{{}_{3}}_{i}}^{{}_{\mathrm{LUX}}}}\right)}^{2}],$$

where the coefficient values of Gaussian functions are identified as $g_{{}_{11}}^{{}_{\mathrm{LUX}}}$ = 0.76, $g_{{}_{21}}^{{}_{\mathrm{LUX}}}$ = 0°, $g_{{}_{31}}^{{}_{\mathrm{LUX}}}$ = 29°, $g_{{{}_{1}}_{2}}^{{}_{\mathrm{LUX}}}$ = 1.10, $g_{{}_{22}}^{{}_{\mathrm{LUX}}}$ = 45°, and $g_{{{}_{3}}_{2}}^{{}_{\mathrm{LUX}}}$ = 21° [7]. Similarly, the 3D display of LUXEON Rebel optical beam and the relevant indoor scenario are shown in Figure 2. Based on the above LUXEON Rebel beam mathematical expression, the MATLAB plot tool is utilized to draw the Figure 2 as well. The strength of the two Gaussian terms is linked to the basic components of LED source (chip, mirror and lens). Although this non-Lambertian beam is still rotationally symmetric, the concerned direction of maximum radiation intensity obviously deviates the normal direction of the source, which means that more optical power is emitted to the coverage edge area [7,8].

**Figure 3:**
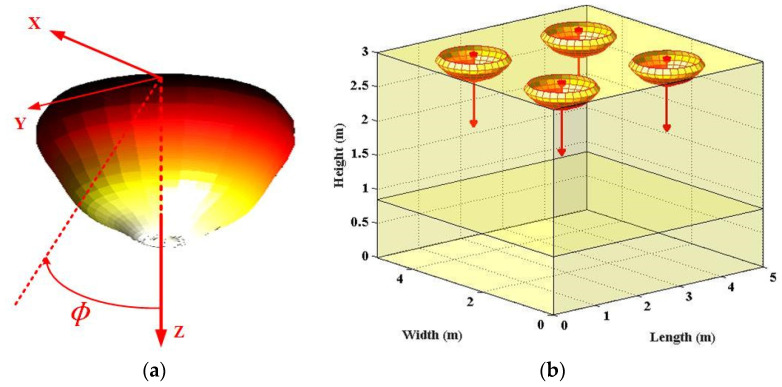
(**a**) 3D spatial optical beam of Side Emitter non-Lambertian LED; (**b**) Side Emitter non-Lambertian LED-applied indoor scenario, respectively.

As for the Side Emitter case, the spatial radiation intensity could be profiled by one sum of three Gaussian functions [7]:

(3) $$I_{\mathrm{SID}}(\phi )=\sum_{i=1}^{3}g_{{{}_{1}}_{i}}^{{}_{\mathrm{Sid}}}\exp[-\ln2{\left(\frac{\left|\phi \right|-g_{{{}_{2}}_{i}}^{{}_{\mathrm{Sid}}}}{g_{{{}_{3}}_{i}}^{{}_{\mathrm{Sid}}}}\right)}^{2}],$$

where the included Gaussian functions coefficients could be identified as $g_{{}_{11}}^{{}_{\mathrm{Sid}}}$ = 0.542, $g_{{}_{21}}^{{}_{\mathrm{Sid}}}$ = 22.75°, $g_{{}_{31}}^{{}_{\mathrm{Sid}}}$ = 49.96°, $g_{{{}_{1}}_{2}}^{{}_{\mathrm{Sid}}}$ = 0.573, $g_{{}_{22}}^{{}_{\mathrm{Sid}}}$ = 77.84°, $g_{{{}_{3}}_{2}}^{{}_{\mathrm{Sid}}}$ = 23.7°, $g_{{{}_{1}}_{3}}^{{}_{\mathrm{Sid}}}$ = 0.279, $g_{{}_{23}}^{{}_{\mathrm{Sid}}}$ = 86.67°, and $g_{{{}_{3}}_{3}}^{{}_{\mathrm{Sid}}}$ = 8.43° specifically [7]. Accordingly, 3D spatial optical beam of Side Emitter non-Lambertian LED and Side Emitter non-Lambertian LED-applied indoor scenario is presented in Figure 3. Respectively, based on the above Side Emitter beam mathematical expression, the MATLAB plot tool is utilized to draw the Figure 3.

## 3. Analytical VLC Channel Model

### 3.1. Lambertian VLC Channel Model

For conventional Lambertian beam configuration, the VLC channel gain includes line of sight (LOS) and non-line of sight (NLOS) components.

For the LOS portion, the channel frequency response could be given as [6]

(4) $$H_{{}_{\mathrm{LOS}}}^{\mathrm{Lam}}(f;T,R)=\{\begin{array}{c} \frac{A_{R}}{d^{2}}I_{\mathrm{Lam}}\left(\phi \right)G_{\mathrm{of}}G_{\mathrm{oc}}\cos\left(\theta \right)e^{-j2\pi f\Delta t_{\mathrm{LOS}}},0\leq \theta \leq \theta_{\mathrm{FOV}}\\ 0,\;\;\;\;\;\;\;\;\;\;\;\;\;\;\;\;\;\;\;\;\;\;\;\;\;\;\;\;\;\;\;\;\;\;\;\;\;\;\;\;\;\;\;\;\;\;\;\;\;\;\;\;\;\;\;\;\;\theta \geq \theta_{\mathrm{FOV}} \end{array},$$

where $A_{R}$ is the physical area of the receiver *R*, *d* is the distance between the optical transmitter *T* and the receiver *R*, $G_{\mathrm{of}}$ is the optical filter gain, $G_{\mathrm{oc}}=n_{\mathrm{RI}}/\left(\sin^{2}\left(\theta_{\mathrm{FOV}}\right)\right)$ is the optical concentrator gain at the receiver *R* with internal refractive index $n_{\mathrm{RI}}$, $\Delta t_{\mathrm{LOS}}$ is propagation delay of the LOS path and is the field of view (FOV) at the receiver.

(5) $$H_{\mathrm{NLOS}}(f;T,R)=\eta_{\mathrm{DIFF}}\frac{e^{-j2\pi f(\Delta T+\Delta t_{\mathrm{LOS}})}}{1+j\frac{f}{f_{0}}},$$

where ΔT is the delay between the LOS component and the NLOS component onset, $f_{0}$ is the NLOS component cutoff frequency, and $\eta_{\mathrm{DIFF}}$ is the power efficiency for the NLOS component [5]. Specifically, $\eta_{\mathrm{DIFF}}$ could be calculated as follows

(6) $$\eta_{\mathrm{DIFF}}=\frac{A_{R}}{A_{\mathrm{ROOM}}}\frac{\langle \rho \rangle }{1-\langle \rho \rangle },$$

where $A_{\mathrm{ROOM}}$ is the entire room surface, and 〈ρ〉 is the average reflectivity of the room surface. The mentioned $f_{0}$ can be identified by $f_{0}=1/\left(2\pi \tau \right)$ where the exponential decay time is given by

(7) $$\tau =-\frac{\langle t\rangle }{\mathrm{In}\langle \rho \rangle },$$

The figure 〈t〉 can be viewed as the average time between two reflections. In typical a rectangular room, 〈t〉 is given as follows

(8) $$\langle t\rangle =\frac{4V_{\mathrm{ROOM}}}{cA_{\mathrm{ROOM}}}=\frac{2}{c}\frac{l\cdot w\cdot h}{l\cdot w+l\cdot h+w\cdot h},$$

where *l*, *w*, and *h* are the length, the width, and the height, respectively [5]. Therefore, under Lambertian optical beam configuration, the whole channel frequency response could be written as

(9) $$H_{{}_{\mathrm{VLC}}}^{\mathrm{Lam}}(f;T,R)=H_{{}_{\mathrm{LOS}}}^{\mathrm{Lam}}(f;T,R)+H_{{}_{\mathrm{NLOS}}}(f;T,R),$$

### 3.2. Non-Lambertian VLC Channel Model

Obviously, unlike the NLOS component, the channel frequency response of the LOS portion is tightly relevant to the optical beam radiation characteristic.

Respectively, for the LUXEON Rebel beam case, the LOS channel frequency response should be given by [7,8]:

(10) $$H_{{}_{\mathrm{LOS}}}^{\mathrm{LUX}}(f;T,R)=\{\begin{array}{c} \frac{A_{R}}{d^{2}}I_{\mathrm{LUX}}\left(\phi \right)G_{\mathrm{of}}G_{\mathrm{oc}}\cos\left(\theta \right)e^{-j2\pi f\Delta t_{\mathrm{LOS}}},0\leq \theta \leq \theta_{\mathrm{FOV}}\\ 0,\;\;\;\;\;\;\;\;\;\;\;\;\;\;\;\;\;\;\;\;\;\;\;\;\;\;\;\;\;\;\;\;\;\;\;\;\;\;\;\;\;\;\;\;\;\;\;\;\;\;\;\;\;\;\;\;\;\theta \geq \theta_{\mathrm{FOV}} \end{array},$$

The whole channel frequency response should be rewritten as:

(11) $$H_{{}_{\mathrm{VLC}}}^{\mathrm{LUX}}(f;T,R)=H_{{}_{\mathrm{LOS}}}^{\mathrm{LUX}}(f;T,R)+H_{{}_{\mathrm{NLOS}}}(f;T,R),$$

Similarly, for the Side Emitter beam case, the LOS channel frequency response is given by

(12) $$H_{{}_{\mathrm{LOS}}}^{\mathrm{SID}}(f;T,R)=\{\begin{array}{c} \frac{A_{R}}{d^{2}}I_{\mathrm{SID}}\left(\phi \right)G_{\mathrm{of}}G_{\mathrm{oc}}\cos\left(\theta \right)e^{-j2\pi f\Delta t_{\mathrm{LOS}}},0\leq \theta \leq \theta_{\mathrm{FOV}}\\ 0,\;\;\;\;\;\;\;\;\;\;\;\;\;\;\;\;\;\;\;\;\;\;\;\;\;\;\;\;\;\;\;\;\;\;\;\;\;\;\;\;\;\;\;\;\;\;\;\;\;\;\;\;\;\;\;\;\;\theta \geq \theta_{\mathrm{FOV}} \end{array},$$

The whole channel frequency response for this beam is written as

(13) $$H_{{}_{\mathrm{VLC}}}^{\mathrm{SID}}(f;T,R)=H_{{}_{\mathrm{LOS}}}^{\mathrm{SID}}(f;T,R)+H_{{}_{\mathrm{NLOS}}}(f;T,R),$$

## 4. MIMO VLC Channel Capacity

### 4.1. Lambertian MIMO Channel Capacity

For one typical MIMO VLC system, the output of the receiver is described by

(14) y=Hx+n,

where **x** = [x_1_, x_2_, …, x*_l_*]^T^ are emitted symbols from the *l* transmitters, **y** = [y_1_, y_2_, …, y*_k_*]^T^ are the received symbols at the receiver with *k* photo detectors (PD), **n** = [n_1_, n_2_, …, n*_k_*]^T^ are the additive white Gaussian noise (AWGN) at the *k* photo detectors and the direct current (DC) channel gain matrix **H** is given as

(15) $$H=\left(\begin{array}{ccc} H_{{}_{\mathrm{VLC}}}(0;T_{1},R_{1}) & \cdots & H_{{}_{\mathrm{VLC}}}(0;T_{l},R_{1})\\ \vdots & \ddots & \vdots \\ H_{{}_{\mathrm{VLC}}}(0;T_{1},R_{k}) & \cdots & H_{{}_{\mathrm{VLC}}}(0;T_{l},R_{k}) \end{array}\right),$$

where $H_{{}_{\mathrm{VLC}}}(0;T_{n},R_{m})$ represents the DC channel gain between the transmitter $T_{n}$ and the photo detector $R_{m}$. Following the previous work, in one well-lit environment, the shot noise is a dominant contributor to signal disturbance at the receivers. Then the noise power could be calculated as

(16) $$N_{0}=2qI_{\mathrm{bg}}B+\frac{4K_{b}TB}{R_{f}},$$

where *q* denotes the electron charge in coulombs, *I*_bg_ is the current due to background light; *B* is the system modulation bandwidth; *K*_b_ denotes the Boltzmann constant; *T* is the absolute temperature and *R*_f_ is the feedback resistance of the transimpedance amplifier (TIA) [11].

Following the work of Harald Haas, the capacity equation for RF MIMO channels can be utilized to estimate the theoretical capacity of MIMO VLC Gaussian channels. Specifically, its formula is represented here for convenience [10]:

(17) $$C_{\mathrm{Lam}}=B\log_{2}\left(\det\left(I+\frac{P_{s}}{N}H_{\mathrm{Lam}}H_{\mathrm{Lam}}^{H}\right)\right),$$

where *B* is the system modulation bandwidth, det() denotes the determinant, **I** is the identity matrix, Ps denotes the emitted signal power at each transmitter, *N* is the total noise variance and []^H^ is the Hermitian conjugate of one matrix [10]. Moreover, following (15), channel matrix **H**_Lam_ could be given for Lambertian optical beam, and the element of this matrix should be calculated by (9) with =0.

### 4.2. Non-Lambertian MIMO Channel Capacity

For non-Lambertian optical beams, the above VLC channel capacity formula must be renewed accordingly. For the LUXEON Rebel beam case, the respective channel capacity is given by [7,8,10]:

(18) $$C_{\mathrm{LUX}}=B\log_{2}\left(\det\left(I+\frac{P_{s}}{N}H_{\mathrm{LUX}}H_{\mathrm{LUX}}^{H}\right)\right),$$

where **H**_LUX_ is the channel matrix for LUXEON Rebel optical beam, and the element of this matrix should be calculated by (11) with f = 0.

Moreover, for the Side Emitter beam, the respective channel capacity is given by [7,8,10]:

(19) $$C_{\mathrm{SID}}=B\log_{2}\left(\det\left(I+\frac{P_{s}}{N}H_{\mathrm{SID}}H_{\mathrm{SID}}^{H}\right)\right),$$

where **H**_SID_ is the channel matrix for Side Emitter optical beam, and the element of this matrix should be calculated by (13) with f = 0.

## 5. Numerical Analysis

For investigating the optical beam effects on VLC channel characteristics, the typical indoor scenario is adopted from the classic publication, which is consistent with the scenario shown in Figure 1b, Figure 2b and Figure 3b. For fair comparison, all three optical beam patterns are normalized within the following work. Furthermore, Table 1 summarizes the main parameters for this work.

**Figure 4:**
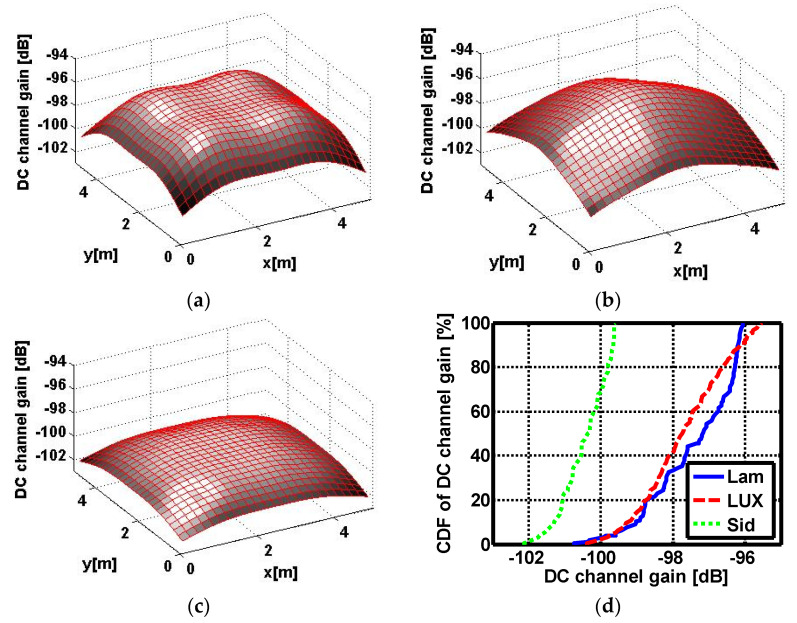
DC channel gains spatial distribution of (**a**) Lambertian optical beam case; (**b**) LUXEON Rebel optical beam case; and (**c**) Side Emitter optical beam case. (**d**) Cumulative distribution function of the above DC channel gains.

In Figure 4, the DC channel gains spatial distribution are shown for the Lambertian and investigated two non-Lambertian optical beams. For conventional Lambertian beam case, the DC channel gain dynamic range is about −100.74~−96.05dB while the counterpart of the LUXEON Rebel optical beam case is about−100.38~−95.51 dB. Due to the distinct radiation pattern for the Side Emitter beam, the respective dynamic range is dramatically reduced to −102.12~−99.62 dB.

**Figure 5:**
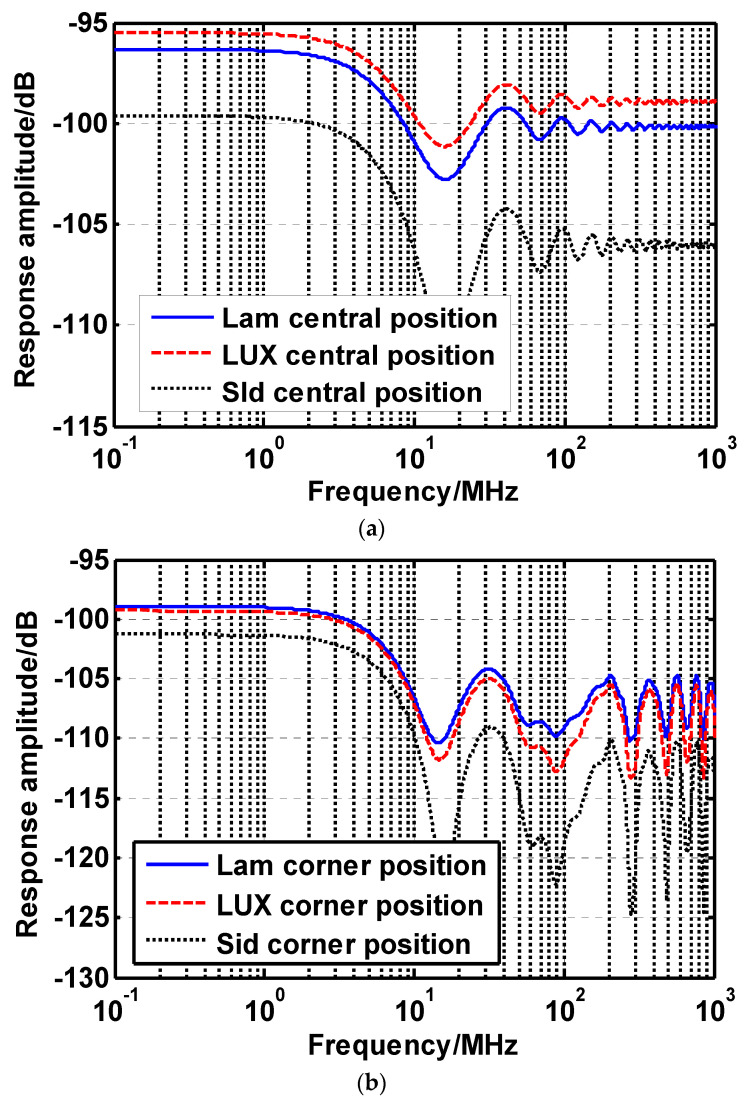
Frequency response curves of (**a**) central receiver position; and (**b**) corner receiver position.

The frequency response curves for the typical receiver positions are shown Figure 5. Specifically, at the central receiver position (2.5 m, 2.5 m, 0.85 m), compared to the conventional Lambertian beam, the LUXEON Rebel beam is capable of providing about 0.82 dB frequency response gain within system bandwidth range. In addition, the Side Emitter optical beam induces up to about 3.29 dB frequency response loss at the same position.

For the corner receiver position (0.5 m, 0.5 m, 0.85 m), the frequency response gain of the LUXEON Rebel beam is slightly weaker than the counterpart of the Lambertian beam. On the other hand, the gain gap between the Side Emitter optical beam and the Lambertian beam is reduced to 2.35 dB. All above results identify that the non-Lambertian beams could reshape the VLC spatial coverage and provide distinct channel gain distribution.

For analyzing the optical beam effects on the MIMO transmission, the MIMO capacity performance is estimated under the concerned three optical beam configurations.

**Figure 6:**
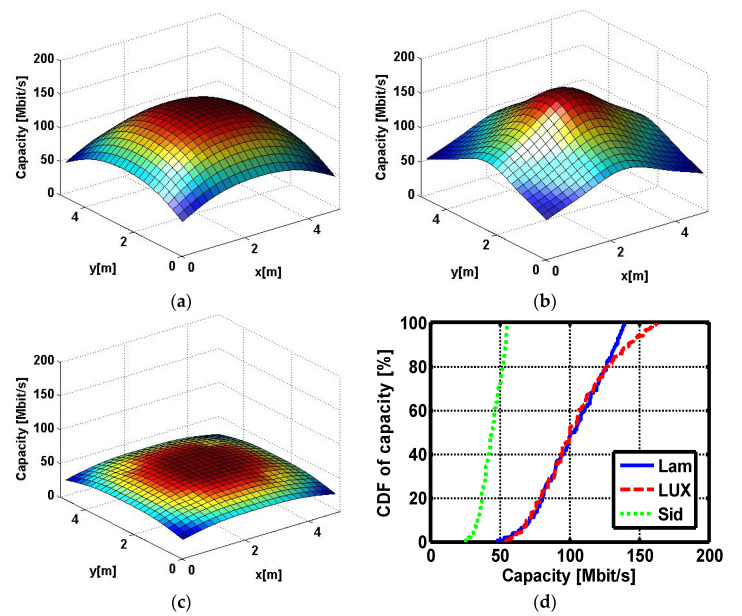
MIMO capacity spatial distribution of (**a**) Lambertian optical beam case; (**b**) LUXEON Rebel optical beam case; and (**c**) Side Emitter optical beam case. (**d**) Cumulative distribution function of the above MIMO capacity.

For the convenience of analysis, at the MIMO receiver, four PD are included, and each is assumed perfectly alignment to one separate optical source. From Figure 6, it could be observed that LUXEON Rebel optical beam could present a similar MIMO capacity level to the Lambertian optical beam, but the peak capacity appears at the solo central position, and not under the four separate optical source positions. For the Lambertian optical beam case and LUXEON Rebel optical beam case, the average capacity is 102.53 Mbps and 103.99 Mbps, respectively. The counterpart of Side Emitter optical beam case is 44.27 Mbps, therefore Side Emitter optical beam could induce up to 58.26 Mbps average capacity degradation with improved performance uniformity. The respective average MIMO transmission capacity deviation is about 56.8% compared to the Lambertian baseline case.

**Figure 7:**
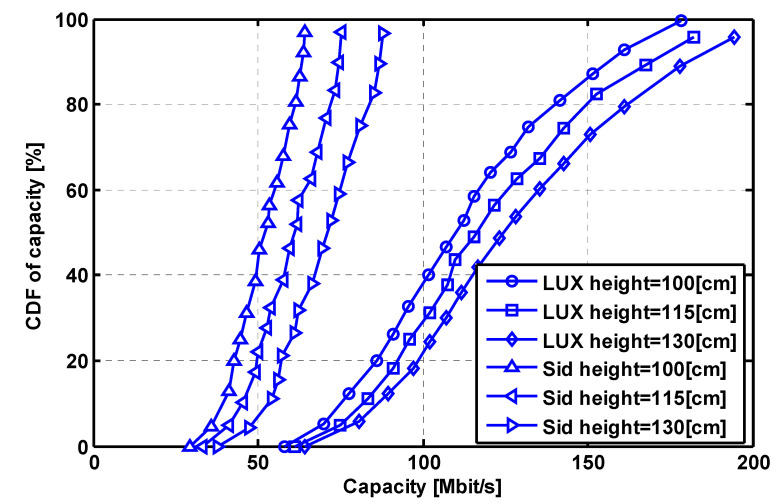
Comparison of the MIMO capacity CDF under different receiver height.

For the conventional Lambertian beam case, the MIMO transmission capacity dynamic range is about 48.27~140.01 Mbps while the counterpart of the LUXEON Rebel optical beam case is about 54.64~164.25 Mbps. As for the left Side Emitter beam case, the relevant dynamic range is dramatically reduced to 25.59~55.13 Mbps. For clear comparison, the Cumulative distribution function (CDF) curves of Lambertian optical beam case and two considered non-Lambertian optical beam cases are described in Figure 6d according to the three distinct MIMO capacity spatial distribution illustrated in Figure 6a–c. For evaluating the MIMO capacity under different receiver height, three height settings, i.e., 100 cm, 115 cm and 130 cm are considered for the concerned two non-Lambertian optical beam cases. In this situation, under three receiver height setting, all six different non-Lamertain MIMO capacity distributions could be identified, and the respective six CDF curves are described in Figure 7. Moreover, as shown in Figure 7, elevating the receiver height could bring a more obvious MIMO capacity gain to the LUXEON Rebel optical beam case than the Side Emitter optical beam case. For LUXEON Rebel optical beam case, the average capacity is 112.53 Mbps for height of 100 cm, and the counterpart is increased to 120.94 Mbps and 129.00 Mbps for receiver heights of 115 cm and 130 cm. On the other hand, similar capacity gain could also be observed by increasing receiver height for the Side Emitter beam case. Accordingly, the average capacity is 51.67 Mbps for height of 100 cm, and the counterpart is increased to 60.25 Mbps and 70.13 Mbps for receiver heights of 115 cm and 130 cm.

## 6. Conclusions

This work has investigated the VLC channel characteristic evolution from the conventional Lambertian beam to typical non-Lambertian beams. The potential beam effects on VLC DC channel gain and MIMO capacity distribution are illustrated for the first time. For the typical corner receiver position, the channel gain gap between the Side Emitter optical beam and the Lambertian beam is still up to 2.35 dB. Under distributed transmitter configuration, the Side Emitter optical beam could provide more uniform capacity distribution at the price of about 56.8% average MIMO transmission capacity deviation, compared to the Lambertian baseline case. On the other hand, the capacity distribution is more sensitive to receiver height for the LUXEON Rebel optical beam.

## Figures and Tables

**Table 1:** Summarizes the main parameters for this work.

Parameters	Values
Room size (W × L × H)	5 ×5 × 3 m^3^
Reflection coefficient of walls	0.60
Emitted power of each transmitter	100 mW
LED Lambertian index	1
Receiver field of view	30°
Physical area of PD	1 cm^2^
Responsively of PD	0.4 A/W
Concentrator refractive index	1.54
Optical filter gain	1
Modulation bandwidth	10 MHz
Charge of an electron	1.602 × 10^−19^
Background light current	5100 μA
Absolute temperature	298 K
Feedback resistance of TIA	6 kΩ

## Data Availability

Not applicable.

## References

[1] Chi N., Zhou Y., Wei Y., Hu F. (2020). Visible light communication in 6G: Advances, challenges, and prospects. IEEE Veh. Technol. Mag..

[2] Emilio C., Sergio B., Jose L. (2019). 6G: The next frontier: From holographic messaging to artificial intelligence using subterahertz and visible light communication. IEEE Veh. Technol. Mag..

[3] Ma X., Gao J., Yang F. (2017). Integrated power line and visible light communication system compatible with multi-service transmission. IET Commun..

[4] Song J., Ding W., Yang F. (2015). An indoor broadband broadcasting system based on PLC and VLC. IEEE Trans. Broadcast..

[5] Jungnickel V., Pohl V., Nonnig S. (2002). A physical model of the wireless infrared communication channel. IEEE J. Sel. Areas Commun..

[6] Komine T., Nakagawa M. (2004). Fundamental analysis for visible-light communication system using LED lights. IEEE Trans. Consum. Electron..

[7] Moreno I., Sun C. (2008). Modeling the radiation pattern of LEDs. Opt. Express.

[8] Ding J., Chih-Lin I., Xu Z. (2016). Indoor optical wireless channel characteristics with distinct source radiation patterns. IEEE Photonics J..

[9] Ding J., Chih-Lin I., Zhang H. (2019). Cells planning of VLC networks using non-circular symmetric optical beam. Proceedings of the 2019 IEEE International Conference on Communications (ICC).

[10] Tsonev D., Sinanovic S., Haas H. (2013). Practical MIMO capacity for indoor optical wireless communication with white LEDs. Proceedings of the IEEE 77th Vehicular Technology Conference (VTC Spring).

[11] Chen C., Tsonev D., Haas H. (2013). Joint transmission in indoor visible light communication downlink cellular networks. Proceedings of the IEEE Globecom Workshops.

